# Extreme Desire for Motherhood: Analysis of Narratives From Women Undergoing Assisted Reproductive Technology (ART)

**DOI:** 10.5964/ejop.v15i2.1736

**Published:** 2019-06-07

**Authors:** Viviana Langher, Fabiola Fedele, Andrea Caputo, Francesco Marchini, Cesare Aragona

**Affiliations:** a Department of Dynamic and Clinical Psychology, “Sapienza” University, Rome, Italy; b Department of Gynecologic-Obstetrical and Urologic Sciences, “Sapienza” University Hospital Umberto I, Rome, Italy; c Sterility and Assisted Reproduction Unit, “Sapienza” University Hospital Umberto I, Rome, Italy; Webster University Geneva, Geneva, Switzerland; University of Neuchâtel, Neuchâtel, Switzerland

**Keywords:** infertility, assisted reproductive technology, emotions, text analysis, women’s narratives

## Abstract

The problem of infertility and its consequent treatment (denoted as Assisted Reproductive Technology or ART) represent an increasing phenomenon, especially in industrialized countries. Confronting with one’s own procreative limitations can generate strong negative emotional reactions. This study aims at understanding how the desire for motherhood manifests itself in infertile women undergoing ART, studying their emotional and subjective perspective. An in-depth explorative research study was conducted on 17 infertile women attending an Italian hospital clinic for fertility treatment. Emotional text analysis was conducted to analyze the corpus of their interviews, allowing the identification of four thematic domains (clusters) which refer, respectively, to the following emotional dimensions: an inclination to self-sacrifice, seen as the price to be paid for the desired success of the treatment (Cluster 1), pursuit of inclusion in the world of procreative mothers (Cluster 2), precarious equilibrium between the deep desire for a baby and the withdrawal from the treatment (Cluster 3), surrender to any possible consequence in order to obtain the desired mother-child relationship (Cluster 4). The witness of the couples’ suffering for their condition of infertility and their strong desire for parenting can represent a source of high pressure for the fertility care staff, as they are the only ones responsible for the fulfillment of the great dream of biological parenthood. For these reasons, a multidisciplinary approach, which involves psychological as well as medical experts all working together, could benefit both the patients and the healthcare professionals and improve the quality of the reproductive healthcare services.

Infertility is a problem afflicting about 186 million people worldwide. On average, 9% of all couples in the reproductive age suffer from this condition. In some regions of the world, specifically where reproductive medicine comes in, infertility rates affect as much as 30% of couples (Stevenson & Hershberger, 2016).

The term “Assisted reproductive technology” (ART) refers to medical treatments used to achieve a pregnancy when it does not occur spontaneously, such as Intrauterine Insemination (IUI), In Vitro Fertilization (IVF), and Third Party-Assisted ART (with gamete donors). Specifically, In Vitro Fertilization is the most common and effective type of ART used in absence of sperm/egg donation from people outside the couple. It consists in removing the eggs from the woman’s body and then mixing them with the sperm to create the embryos that are then put back into the woman’s uterus. Since the first *in vitro* baby in 1978, the number of people conceived by reproductive technology has grown much faster than expected, reaching several million today and rapidly approaching 0.1% of the total world population (Faddy, Gosden, & Gosden, 2018). In 2014 alone, the number of children born in Italy through the application of assisted reproduction techniques constituted 2.5% of the total newborns that year. Data from the National Registry of Medically Assisted Reproduction from 2005 to 2014 confirm the presence of a growing trend, with percentages of births with ART techniques constantly increasing (Scaravelli, Vigiliano, De Luca, Fiaccavento, & Speziale, 2014). The spread of the problem of infertility suggests the need to promote the widespread knowledge and improve the public awareness of this health issue (Place, Litwack, & Vann, 2018).

## The Subjective Experience of Infertility

As various research shows, coming to terms with one’s own procreative limitations could generate strong negative reactions such as anger, pain, dejection and frustration (Fassino, Pierò, Boggio, Piccioni, & Garzaro, 2002; Galhardo, Cunha, & Pinto-Gouveia, 2011; Vitale, La Rosa, Rapisarda, & Laganà, 2017). The condition of infertility could also be associated with a deep sense of guilt, shame, and decreasing self-esteem (Yao, Chan, & Chan, 2018). Diagnosis of infertility could produce, indeed, a self-deprecating sense of feminine or masculine insufficiency. The concerns of people with the problem of infertility seem to relate to a sense of personal disappointment and guilt due to failing “essentially to do what you’re put on earth to do” (Schofield, 2014). Other studies show that discovering one’s infertility could generate feelings of impotence and loss of control over one’s life (Benyamini, Gozlan, & Kokia, 2005; Cousineau & Domar, 2007) and one’s own body (Clarke, Martin-Matthews, & Matthews, 2006). People with infertility problems also often manifest feelings of self-criticism and social isolation (Batista, Bretonesa, & De Almeida, 2016; Cunha, Galhardo, & Pinto-Gouveia, 2016). As far as the perception of isolation is concerned, some researchers have focused on the feelings of envy and jealousy towards relatives or friends who, on the contrary, do have children. These reactions could possibly cause the couple to interrupt these relationships, thus increasing their feeling of isolation (Mann, 2014).

Failing to procreate also means having to face the mourning of the desired imaginary child and having to confront oneself with the loss of one’s own procreative function. Infertility could generate a deep narcissistic wound in the individual, which could in turn compromise self-image with respect to one’s own gender and generative identity (Galasso, Langher, & Ricci, 2017).

Infertility could also have a considerable impact on the marital life of the couple, triggering some psychological difficulties, including: lack of marital satisfaction, impairment of relationships, lack of sexual satisfaction, forced timing of intercourse, loss of confidence in relation to sex, decreased libido and negative emotional effects (Ashraf, Ali, & Azadeh, 2015; Cousineau & Domar, 2007).

Especially in certain societies, becoming a mother is almost a cultural mandate, according to which society and families naturally expect a young woman to eventually give birth to a child. The scientiﬁc literature on infertility is increasingly emphasising the importance of the sociocultural context in shaping the lived experience of infertility. Therefore in cultural contexts in which voluntary childfree status is acknowledged, many women experience infertility as a “secret stigma” (Greil, 1991); in cultural contexts in which there is no concept of voluntary childfree status, it is impossible to hide infertility. The stigma and distress of infertility, therefore, is likely to be greater (Dyer, Abrahams, Mokoena, Lombard, & Van der Spuzy, 2005). Moreover, as several studies have shown, infertile women who experience rejection or pressure from husbands and family experience higher levels of distress and suffering (Gulseren et al., 2006; Guz, Ozkan, Sarisov, Yanik, & Yanik, 2003)

Still today, therefore, motherhood is often considered to be a primary role for women; as well, women who do not mother either biologically or socially are often stereotyped as either desperate or selfish (Letherby & Williams, 1999). The stereotypes of non-motherhood are still present: the “infertile” and “involuntarily” childless women are desperate and unfulfilled (Franklin, 1997; Letherby & Williams, 1999) and the “voluntarily” childless ones are viewed as selfish and deviant and portrayed in ways that emphasize them as aberrant, immature, and unfeminine (Gillespie, 2000). For these reasons, women can feel a huge amount of emotional pressure to procreate (Holmes, 2014).

## The Experience of Undergoing Assisted Reproduction Technology

People who come to terms with one’s own infertility could decide to resort to assisted reproduction techniques. The experience of infertility treatment has been described as a situation that engulfs patients and dominates their daily routine (Daniluk, 2001; Redshaw, Hockley, & Davidson, 2007). Furthermore, people who resort to ART often are not aware of the fertility decline related to the maternal age (especially after 35 years old), overestimating success rates of these techniques with unrealistic expectations (Hayward, 2016). In fact, many therapeutic attempts are often necessary before a child can be conceived, and, despite all the efforts, sometimes this does not even happen. It should be acknowledged that even if the ART path is successful and leads to the birth of a healthy child, the representation of one’s own fecundity could remain equally compromised. The pain caused by the infertility could remain, in fact, even after the success of the reproductive techniques. The narcissistic wound of “not having done it alone” may never be completely healed, thus undermining the image of oneself and one’s Ego Ideal (Agostini et al., 2009). Conceiving a baby *through ART*, in brief, does not cancel the presence of the sterile sexuality, but it becomes the proof of such a sterile sexuality.

Furthermore, during the course of ART treatment, the sense of dependence on the doctor, both in a physical and psychological sense, may become prominent. The patients’ feeling of *helplessness* may in fact resemble the feeling of *impotence* of a child towards his/her own parents, promoting an intense emotional bond of the patient with the doctor (Cudmore, 2005). This could represent a critical moment for the couple, due to the intrusion of the medical team and its procedures in the intimate life of the couple. Undergoing an ART procedure is also associated with the presence of significantly higher depression and sense of shame levels than those obtained by fertile or infertile couples not going through ART (Galhardo, Pinto-Gouveia, Cunha, & Matos, 2011). In fact, ART patients may experience the procedure as a source of deep stress, anxiety and concern (Brandes et al., 2009). Levels of anxiety and depression increase, moreover, with the progression of therapeutic failures, generating a real “failure syndrome” (Verhaak et al., 2005).

Greil (2002) summarizes the experience of treatment of patients with infertility problems in terms of three paradoxes: 1) their sense of loss of control leads them to treatment where they lose even more control; 2) their feelings of loss of bodily integrity leads them to treatment where the body is invaded; and 3) their sense of loss of identity leads to treatment where they feel they are not treated as whole people.

Although it is difﬁcult to stop treatment, Verhaak et al. (2007) say that stopping treatment leads to reduced depression and anxiety among IVF women, even if they do not conceive. Unsuccessful IVF couples do not regret the IVF experience; instead, they view it as their best chance to have conceived (Daniluk, 2001; Johansson & Berg, 2005; Throsby & Gill, 2004).

The results of the aforementioned research studies describe strong negative emotions associated with the condition of infertility and the appeal to ART treatment. Based on these considerations, we have decided to deeply explore the unconscious emotional dynamics occurring in infertile women. Getting in contact with these emotional dynamics allowed us to make some hypotheses about the possible practical and behavioral implications concerning women with infertility problems, especially those undergoing Assisted Reproductive Technology. We have chosen, therefore, not to refer to well-defined and a priori emotional indicators, that is, depressive symptoms or trait or state anxiety, as in the case of the aforementioned research studies, but rather to formulate data-driven hypotheses by using the patients’ narratives. In fact, narrative methods have some advantages over self-report methods, including: reducing social desirability bias, identifying a series of nuances of a given behavior or event that could not be differently understood, and extracting more accurate interpretations based on data (data-driven hypothesis).

## Method

### Participants and Recruitment

The recruitment of participants took place at the fertility clinic of the public healthcare institution “Sapienza” University-Hospital Umberto I, Rome (Italy). Eighteen women accessed the clinic during the recruitment phase, matching the following criteria: 1) actively attempting to have a child for at least 1 year; 2) being treated in the hospital clinic for an *In Vitro* fertility procedure; 3) not being at the fertility clinic to seek a pre-implantation genetic diagnosis; and 4) not being diagnosed with psychiatric disorders. Seventeen women who were undergoing In Vitro Fertilization (IVF) gave their informed consent, and only one refused to participate. Nine women were between 28 and 39 years old, the other eight were between 40 and 44. Almost all of them were Italian nationals (*n* = 15), 13 women had low education levels (not achieving beyond upper secondary education) while only 11 were employed. About half of the patients were at their first therapeutic attempt (*n* = 9) while the other half had already made failed attempts and was trying for the second or third time (*n* = 8). Only two patients already had children. The causes of infertility were male factors in six cases, female factors in eight cases, and unexplained (i.e., ascribable to either anatomic or functional issues of either partner) in three cases.

All these different (anamnestic and sociodemographic) variables (called “illustrative variables”) that characterize the participants were analysed in the subsequent phases of textual analysis in order to explore the relationship between these subject-defining characteristics and the emerging thematic domains. The different interview questions were also included as illustrative variables. The interviews were conducted during patients’ waiting times, avoiding interference to clinical practice.

### Instruments

A structured interview—created ad hoc for this study—was administered to infertile patients. The purpose of the interview was to explore the patients’ deep feelings about this particular period of their lives through 12 questions, based on the following three broad categories: 1) the decision to seek treatment and the perceived social support; 2) the emotional perception of the treatment and its effects; and 3) the expectations about the future and the possible pregnancy. The questions asked to the patients were designed *ad hoc* and were formulated after conducting a comprehensive search of the academic literature on the PsychINFO database on the topics of infertility and reproductive techniques, and after a detailed examination of the most frequently mentioned topics in several online forums of ART patients.

All interviews were conducted in person and anonymously (the patients’ names were removed from the transcripts of the texts) as well as audio recorded with permission. The patients have always been encouraged to talk freely about the way they felt and to say everything that came to their mind so to facilitate associative processes. The interviews lasted from a minimum of 10 to a maximum of 40 minutes, with an average time of about 25 minutes. The interviews were conducted from April to July 2017.

### Textual Data Analysis

#### Research Framework

Emotional Text Analysis (ETA) (Carli & Paniccia, 2002; Carli, Paniccia, Giovagnoli, Carbone, & Bucci, 2016) is a psychological tool that aims at exploring people’s affective symbolizations about a shared context, theme or experience, already used in previous research (Caputo, 2014, 2018; Caputo, Giacchetta, & Langher, 2016).

This methodology relies on a psychoanalytic theory of language (Fornari, 1976) assuming a “double reference” principle, according to which the written or spoken language has two functions: lexical-cognitive (conscious) and symbolic-affective (unconscious). The first is responsible for the logical-rational-syntactic structure of language and refers to the sharing of meanings conveyed by the source culture; the second one refers to the figurative meaning based on more symbolic and subjective processes. It controls the expressive components of the lexicon, the juxtaposition of words, the redundancy of terms, and the co-occurrence of terms within discursive units. The “double reference” principle is consistent with Matte Blanco’s bi-logic theory of mind (1981). According to Matte Blanco, in fact, the human mind is able to work in parallel to two different mental processes through which it categorizes the reality: symmetric and homogenizing (or “unconscious”) logic and asymmetric and dividing (or “conscious”) logic. Usually these two ways of being of the mind operate in a parallel, concomitant and intertwined way. According to Matte Blanco, therefore, language can be conceived as a rational instrument addressed to organize reality (as asymmetrical function) but also the gateway to grasp the rules of the mind’s unconscious mode of being (revealing the symmetrical logic). Along with explicit meanings consistent with intentional structuring or ordered constituent parts of language, implicit and symbolic meanings may be inferred from the syntagmatic relations between parts of language. In this regard, the psychoanalytic technique of free association (Freud, 1900), according to which spontaneously associated words and expressions give information on unconscious emotional processes, is an example of how affective sense-making processes can emerge through the logic of association as a form of unconscious thinking. With the same logic with which the analyst interprets the emotional meaning of the patient’s language in his free flow through the analysis of the association of his words, so the researcher can identify the emotional meaning of the associative chains in the speeches of the interviewed subjects.

Moreover, ETA hypothesizes an isomorphism between the co-occurrences statistical calculation and the associative processes characterizing the unconscious mode of thought (Carli & Paniccia, 2002). This method deconstructs the typical linguistic links of the dividing and asymmetrical way of the mind (operational function of language) in order to detect the spontaneous chains of associations between words. Indeed, meaning may be conceived as a property of word combinations rather than as resulting from words by themselves. Besides, to grasp unconscious meanings the concept of polysemy is relevant in terms of emotional meanings attributable to a word, when it is extracted from the linguistic context. According to their polysemic value, the words organizing the language can be divided into two broad categories: dense words, with the maximum of polysemy and the minimum of ambiguity with respect to a contradictory emotional configuration, capable of conveying a high emotional value independently of the linguistic context within which they are located (i.e., words like “bomb” or “good”); and non-dense words, with the maximum of ambiguity and the minimum of polysemy which give meaning to the text only on the basis of their organizing function (for example, articles, adverbs, conjunctions, auxiliary verbs) or ambiguous words that find meaning only through their relationship with other words within the sentence (i.e., words like “to presume” or “however”). When dense words with a high emotional meaning are identified in a text, they can be grouped based on their statistic co-occurrence, thus allowing getting different symbolic repertoires (Caputo, 2013a).

#### Analysis Procedures

Consistently with ETA framework, computer-aided content analysis was used to detect the main symbolic domains emerging from the narratives of the patients with infertility problems. To this purpose, all the interviews were transcribed verbatim and combined in a single text corpus so to detect shared emotional processes. Then, two statistical multidimensional techniques were performed through the T-Lab software, by using the tool “Thematic analysis of elementary contexts” (Lancia, 2004): Cluster Analysis and Multiple Correspondence Analysis. Such analyses elaborate the textual corpus according to a digital “presence-absence” matrix, where rows and columns indicate the parts of discourse to be grouped (i.e., text segments as elementary context units) and the partitioning variables (i.e., words as lexical units) used, respectively. The Cluster Analysis allows the detection of groupings of words co-occurring in the same text segments with the highest probability (i.e., elementary context units), as indicated by the chi-square test (χ²). Such groupings thus represent different semantic classes that can describe the chains of symbolic associations running through language, which progressively contribute to identify cores of meaning across collected interviews with specific regard to the experience of being a patient with infertility problems at an ART centre. Each cluster can be described through the lexical units (dense words) and the most characteristic context units (text segments) from which it is composed of.

Then, Multiple Correspondence Analysis enables the exploration of the relationship between different clusters in a multi-dimensional space. It allows the analysis of the latent factors that organize the main semantic oppositions in the text corpus (regarded as axes in a Cartesian plane representation of this multi-dimensional space) and synthesize the semantic relationships between these groupings (Greenacre, 1984). The relationship between the detected factors and clusters is evaluated through the Test Value, a statistical measure with a threshold value (2), corresponding to the statistical significance more commonly used (*p* = .05) and a sign (-/+) which helps in the understanding of the poles of factors detected through Correspondence Analysis (Bolasco, 1999).

The interpretative process of each cluster and factor (labeled by the researcher) is aimed at giving meaning to the co-occurrence of words of each semantic class and to the contraposition between such semantic classes, based on the use of models of affective symbolization (Carli & Paniccia, 2002). For example, we can identify inclusion/exclusion, power/dependence, trust/mistrust as symbolic dichotomies that have a clear reference to the body and refer to the primitive emotions that people use to bring reality back to something familiar. Such dichotomies seem to reveal different motivational dynamics of social relationships—affiliation, power and achievement—as general affective drives connoting one’s emotional experience and orienting the relations with others consistently with McClelland’s theory of needs (1985). These dynamics were indeed revisited by Carli and Paniccia (2002) and further developed in psychosocial research (Caputo, 2013b, 2019; Langher, Brancadoro, D’Angeli, & Caputo, 2014), within a perspective stemming from constructivism and object relations theory as theoretical foundations. In more detail, the inside/outside dichotomy describes an emotional inclusion/exclusion dynamic. What is “inside” in fact is experienced as something good and friendly, while what is “outside” is perceived as dangerous and rejected. The underlying motivation refer to affiliation as the tendency to establish, maintain, or restore a positive affective relationship with others, accompanied by the fear of rejection. The high/low dichotomy instead describes the symbols of dominating or subjugating in terms of what is perceived as “high” and powerful and what is perceived as “low” and weak. The underlying motivation refer to power, intended as the concern about having status and controlling the means of influencing others’ attitudes, emotions or behaviors, triggered by the fear of losing control and prestige. Then, the in front/behind dichotomy represents the emotional dynamics of the true and the false in terms of showing and concealing. What is “in front” can be seen, reached and verified, differently from what is hidden “behind.” The underlying motivation is achievement, which deals with the disposition to strive for success in accordance with a standard of excellence and to derive satisfaction from the mastery of challenging tasks in order to avoid failure. Such dichotomies represent only some examples of interpretative categories that can be used to grasp the unconscious processes running through discourses. Indeed, the entire interpretative process relies on a psychoanalytic method following an evidential and conjectural paradigm, according to a clinical case study perspective (Langher, Caputo, & Martino, 2017).

The interpretative process is integrated with an in-depth qualitative analysis of the text segments of the interviews (the elementary context units) grouped in each cluster. This allows a process of triangulation and semantic validity of data through cross verification from different sources and procedures, so to get an overall comprehension of the narratives produced by the participants. Some of these text segments have been translated into English and added to the description of each cluster.

## Results

The whole text corpus is of small-medium size (Bolasco, 1999): the total number of linguistic units is between 15,000 and 45,000 occurrences (*N* = 31,758). The indices of lexical richness show a relationship between distinct graphic forms (*V* = 3.323) and total number of occurrences (Type / Token Ratio) equal to 10%. The share of hapaxes (i.e., terms that appear only once in the total amount of distinct graphic forms) is 48% (*N* = 1.628). The admissibility criteria provided by the literature (percentage of hapax <50% and Type/Token Ratio <20%; Bolasco, 1999) are therefore satisfied, allowing us to proceed with a statistical analysis of the text corpus.

Cluster analysis has detected four thematic domains, as shown in Table 1. It should be acknowledged that the detected clusters represent different semantic classes as symbolic meanings emerging across the collected interviews and do not refer to groupings of participants. Therefore, even if such clusters may characterize each interview to a different extent, their related emotional symbolizations are simultaneously present as interacting dynamics in all the produced discourses.

**Table 1:** Clusters, Percentage of the Text Corpus Each Cluster Is Composed of, and a List of the Most Characteristic Lemmas (Keywords)

Cluster 1: Feeling of Self-Sacrifice (21.95%)		Cluster 2: Pursuit of Inclusion (26.72%)		Cluster 3: Between Desire and Withdrawal (20.61%)		Cluster 4: Acceptance of Any Consequence (30.73%)	
Lemma	χ^2^	Lemma	χ^2^	Lemma	χ^2^	Lemma	χ^2^
Goal	20.38	Close	20.50	To leave	63.57	To catch	16.16
Pleasure	20.05	Couple	15.56	To stretch	35.27	To born	15.99
To answer	17.39	Family	13.40	Psychology	33.93	To decide	15.44
Different	13.84	World	12.14	Sister	29.99	Follicle	13.78
To imagine	11.40	Person	11.56	Physical	29.83	Ovary	11.31
Patience	11.33	Colleague	11.05	Woman	29.97	First time	11.31
Health	10.69	Technique	9.67	Brother	22.21	Institute	11.21
To face	9.40	To approach	7.32	To lose	20.78	Normality	11.21
To hope	9.19	Friendship	6.80	Emotion	20.01	Me	11.04
To believe	8.88	Honesty	6.80	Reality	13.64	Husband	8.03
Stress	7.43	To support	4.94	To comprehend	12.71	Loneliness	6.83
Future	6.97	Sorrow	4.94	To move on	11.19	Doctor	5.55
To struggle	4.68	Intimacy	4.79	To desire	6.30	Guilt	5.52
God	4.68	To sustain	4.44	To need	5.78	Son	5.26
To overcome	4.68	Truth	3.98	Pain	4.78	Mother	4.71
Step	4.26			Longing	4.28	Tube	4.39

### Thematic Domains

#### Cluster 1: Self-Sacrifice Seen as the Price to Be Paid for the Desired Success of the ART Treatment

This cluster includes 21.95% of elementary context units. This thematic domain describes the willingness of patients to endure all the efforts and fatigues that the ART treatment requires in order to achieve the desired purpose: motherhood. The suffering caused by undergoing the treatment is felt by these women with great dedication and patience (“patience,” “to overcome”). The sense of self-sacrifice seems to be prevalent in this theme as it leads to the hoped maternity, hence making it possible to achieve social and personal approval. The ART treatment is evoked as consisting of steps to be passed (“step”), symbolized by the patients as huge obstacles to be overcome (“stress,” “to struggle,” “to overcome,” “to face”). The confident expectation that these obstacles will be overcome and will lead to the desired goal sustains these women in their efforts (“to hope,” “to believe”) and comfort them as if it were a sort of prayer (“God”).

*It's a stressful situation; however, you have to hold on because you have to reach a goal. As well, you have to be very strong and totally comply whit what they [doctors] tell you. I feel stressed, I cannot hide it*. (Patient 16)

The illustrative variables that characterize this cluster are: “presence of an employment,” “first therapeutic cycle,” “high educational level,” and “perspectives about the future.” Having a job and a high level of education may represent significant personal resources, increasing patients’ confidence in the ability to succeed in their actions. This in turn makes them more confident in the treatment success.

The association with the questions about the future and the condition of being on their first ART cycle also explains for the women’s perspective of a future filled with hope, since they have not yet experienced the fallibility of reproductive techniques on their own body.

#### Cluster 2: Pursuit of Inclusion

This cluster includes 26.72% of elementary context units and is not associated with any illustrative variable. This thematic domain describes the ambition of ART patients to belong to the world of fertile and generative mothers, from which they feel excluded due to their infertility condition. This cluster seems to describe the pursuit for closeness and identification with the symbolic community of procreative mothers (“close,” “couple,” "family"), that they aim to join with by removing the sterility, which makes them different from the procreative mothers.

*To be honest it is a bad situation because I am now at an age where I meet a lot of people who begin having babies. In fact, right now I have four pregnant colleagues at work, and that it's ugly. For so long I have been trying, taking medicines, following treatments. Sometimes it’s heavy, difficult, very difficult. And then one tries to go on, not to think of that, and fell jealousy, but sometimes one is jealous, and you say but why them and not me?* (Patient 12)

The feelings of uncertainty and anguish associated with the sense of exclusion from the generative mothers (“sorrow”) seem emotionally mitigated thanks to the support and closeness of their loved ones (“friendship,” “to support,” “to sustain,” “intimacy,” “family”).

*Certainly my husband gives me a lot of support and understanding. We support each other*. (Patient 6)

#### Cluster 3: Precarious Equilibrium Between Desire and Withdrawal

This cluster includes 20.61% of elementary context units. This theme describes the maximum level of uncertainty in women who are faced with the key dilemma of either continuing to hope for success or completely withdrawing from the treatment. In brief, they seem to be suspended at the apex of their desire for motherhood.

The intense drive to achieve the goal of motherhood (“to stretch,” “to desire,” “to need,” “longing”) emerges in this cluster. Patients are completely focused on what attracts them (the expected baby), sustained by the strong will and determination to obtain it.

*I have dedicated all myself to this [motherhood] and I hope that this beautiful thing happens. At the moment, inside me I feel like it has already happened […]. Oh God, of course, I imagine it will be so beautiful*. (Patient 1)

At the same time, they seem to show also feelings of loss and contemplation about the possibility of having to give up the dream of biological motherhood (“to lose,” “to move on,” “to leave”).

*Well, I’ll assume that at forty someone doesn’t want me to have another baby. Maybe I'm not ready, maybe now I should start enjoying life more, because I've sacrificed all myself for my [first] daughter, so now that she is eleven years old, I could start my life again. So, maybe, I’m destined not to have this baby*. (Patient 13)

In this condition of uncertainty between desire and renunciation, the reference to strong and indissoluble fraternal bonds (“sister,” “brother”), seems to evoke the feeling of belonging: even if they do not have children, they will be always members of their family of origin.

The illustrative variables that characterize this cluster are: “more than one therapeutic attempt,” “unexplained causes of infertility” and “age over 40 years.” These conditions diminish the success rate of the techniques, filling these women with uncertainty and anguish. At the same time, the unexplained causes of infertility would favor the stalled condition in which patients are found. Unexplained infertility may represent, in fact, an incentive to keep desiring biological motherhood, since no concrete obstacles to procreation are found in one’s own or in the partner’s body.

#### Cluster 4: Surrender to any Possible Consequence

This cluster includes 30.73% of elementary context units. It describes the patients’ acceptance of endangering themselves and their romantic relationship to achieve the desired mother-child relationship, which they do not intend to give up, whatever the price they have to pay. In other words, the mother-child relationship absorbs and replaces the relationship with the partners. These women also seem to experience a feeling of body fragmentation, listing single parts of their reproductive organs (“follicle,” “tuba,” “ovary”). Furthermore, the intimate nature of the procedures and the medical attention that these women receive on their bodies can lead them to experience unconscious feelings towards the physicians, as happens in psychotherapy. Specifically, by “transfer” we mean the unconscious displacement of feelings, whether negative or positive, related to significant people of one’s life towards the figure of the health professional (Etchegoyen, 1990). These intense feelings towards the physician could make them put aside the husband emotionally. Feelings of guilt could thus emerge from these women, as they symbolically betray the husband and replace him with a specialist who can inseminate them and who is allowed to "fragment" their body, in exchange for the desired baby (“loneliness,” “guilt”). Guilt and loneliness are therefore two feelings that these women seem to endure in order to obtain that much-desired mother-child relationship.

*I suffered a lot in my life, so I think a child can change me a lot, give me so much and grow up as a person also through the mother-child relationship. I didn’t have a relationship with my mother, and I hope to establish a relationship with my child, to be able to give him/her what is needed, the things I didn’t have in my childhood*. (Patient 2)

The illustrative variables that characterize the cluster are: “absence of an employment” and “female causes of infertility.” The absence of an employment could explain the high emotional investment of the women in the outcome of the procedure, as a possible source of self-realization. Therefore, we understand the patients’ predisposition towards this path despite the physical, emotional and relational costs. Furthermore, the infertility in women could increase their feeling of guilt, for being the reason the couple has to undergo treatment and its negative interferences with the couple relationship.

### Latent Factors

Correspondence Analysis has detected three latent factors, which organize the main semantic oppositions in the text corpus from the different positions of the detected clusters in the factorial space.

Table 2 shows the proportional variance of each factor, explained by each of the four clusters. These three latent factors explain all the data variance (*R*² = 100%). The sign reported in brackets (-/+) indicates the specific factorial pole (negative/positive) associated with each cluster.

**Table 2:** Proportional Variance of Each Factor

Cluster	Factor		
	F1: Motherhood desire	F2: Controversial sense of belonging	F3: Meanings of relationships
Cl1: Feeling of self-sacrifice	0.0042 (-)	**0.9317 (+)**	0.0642 (+)
Cl2: Pursuit of inclusion	0.0101 (+)	**0.4505 (-)**	**0.5930 (+)**
Cl3: Precarious balance	**0.8706 (-)**	0.0296 (-)	0.0998 (-)
Cl4: Surrender to any possible consequence	**0.7239 (+)**	0.0131 (-)	**0.2630 (-)**

*Note.* The sign reported in brackets (-/+) indicates the specific factorial pole (negative/positive) associated with each cluster. The highest associations between clusters and each factor are indicated in bold.

**Figure 1:**
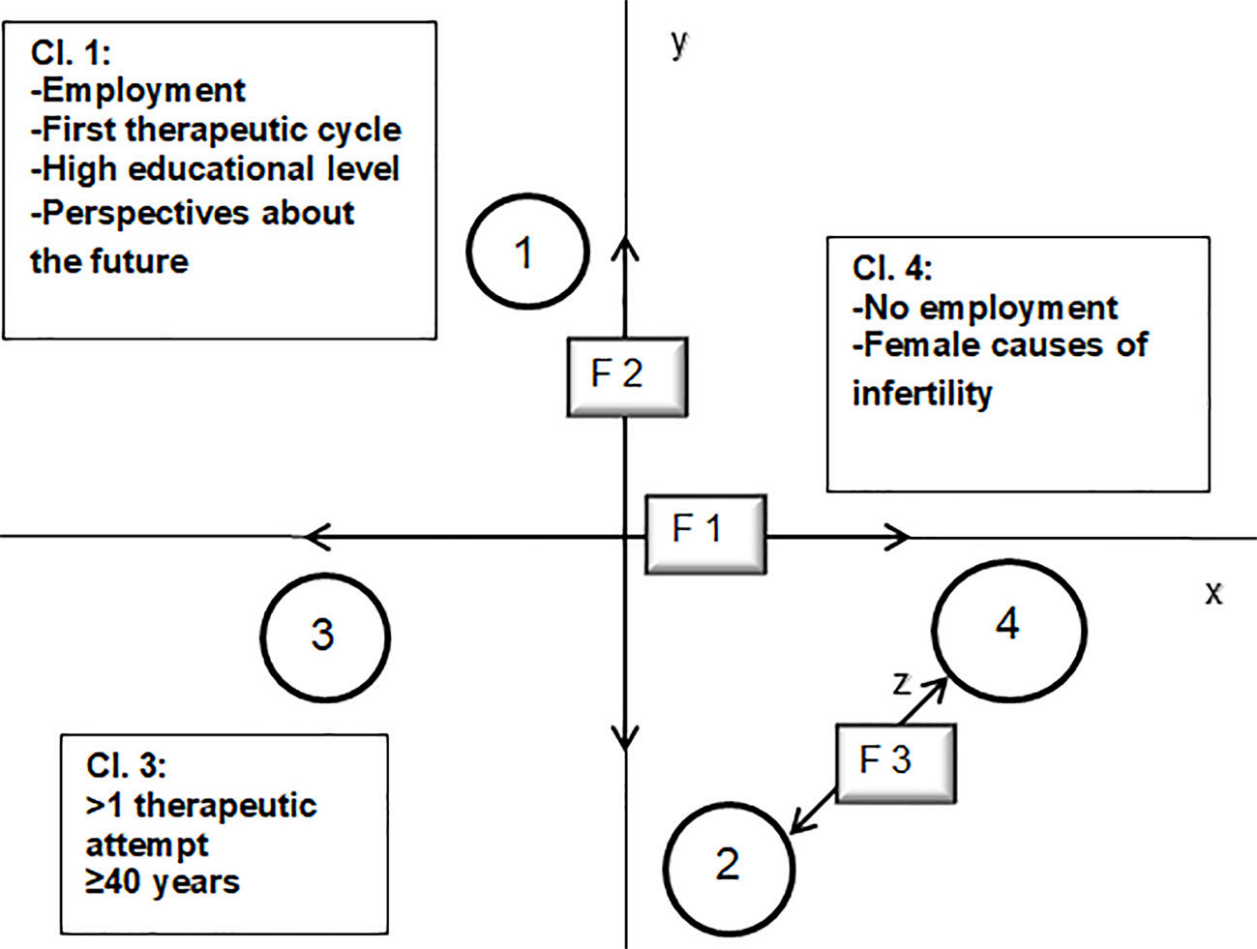
Factorial space.

Figure 1 represents a spatial organization of the clusters within a factorial space and their relationships with the factorial axes. It provides a map of the emotional symbolizations shared by the women interviewed.

#### Motherhood Desire (F1)

The first factor (45.67% of total variance) mainly differentiates Cluster 4 from Cluster 3. It seems to refer to the presence of an intense desire for maternity. On the one hand, it represents the potential withdrawal of desire in terms of giving up the dream of biological motherhood (Cluster 3); on the other hand, the most violent and strong-willed aspects of maternal desire, which could lead the woman to endanger her health and her relationship with the partner in order to reach the desired child (Cluster 4).

#### Controversial Sense of Belonging (F2)

The second factor (33.04% of total variance) differentiates Cluster 1 from Cluster 2. This factor seems to describe the patients’ aspiration to enter into the parental world, from which they feel excluded. Cluster 1 refers to the aspiration to motherhood accompanied by a feeling of confident expectation. For this reason, all the efforts and difficulties of the ART treatments are tolerated. On the opposite pole, in Cluster 2, instead, the feelings of uncertainty and anxiety due to the sense of exclusion from the generative mothers prevail.

#### Meanings of Relationships (F3)

The third factor (21.24% of total variance) differentiates Cluster 2 from Cluster 4. It seems to deal with two different ways in which women live their relationships with others during the ART treatment. On the one hand, their relationships are perceived as a source of support and comfort relatively to their distressing condition of infertility (Cluster 2); whereas, on the other hand, their relationships seem to represent a mere means to achieve their personal goal of motherhood (Cluster 4).

## Discussion

The results of this research study allowed the identification of the main emotional dimensions shaping the experiences of women who undergo an ART treatment. Briefly, the analysis identified four thematic domains (clusters) that describe the intense, profound, painful, and also extreme and radical desire for maternity in these patients with infertility problems. Indeed, this study has revealed the women’s predisposition to bear all the efforts and sufferings that the medical protocol entails in order to achieve motherhood dream. At the same time, these patients have showed a confident perspective, sometimes a real leap of faith, that the motherhood, after so much pain, would come (Cluster 1). In agreement with Castellano et al. (2011), our study have showed that these patients tend to emotionally attribute to the physician and to the ART technique the symbolic meaning of omnipotent restorers of their biological integrity. Indeed, they seem to hopefully expect to be finally saved from their excruciating condition of infertility. With the modern technological innovations people expect to no longer have to suffer the uncertainties of nature but to be able to control them. The shared public perception is, therefore, that doctors can cure infertility, although this is often not possible (Pfeffer, 1993).

Along with patients’ confident desire to belong to the community of procreative women, they have also expressed the fear and anxiety of being excluded from it, failing to fulfill one’s womanliness through motherhood. As we already know, the sense of exclusion and isolation often afflicts the patients with infertility problems (Cunha et al., 2016; Mann, 2014). Moreover, we think that the ambivalence about motherhood was more socially acceptable before the advent of assisted reproductive technologies. Ironically though, the increased availability of reproductive techniques to assist childless people has likely added pressure on infertile women (and men), becoming more reticent to give up on the procreation dream (Letherby, 2002).

To deal with the painful sense of exclusion, the women seem to seek emotional support and comfort in their loved ones in order to make their dramatic condition more tolerable (Cluster 2). Because of this, the possibility of telling and sharing the one’s own condition with someone seems to be beneficial for patients, reducing their sense of isolation.

Therefore, the patients show an intense drive to achieve the goal of motherhood, attempting to get what they do not possess but strongly desire (Cluster 3). Accordingly, the third thematic domain suggests that the women tend to experience a constant state of uncertainty about the future. Their desire for motherhood seems to reach its apex, a point where the women find themselves almost on the verge of renouncing to the great dream of biological motherhood. Consequently, feelings of dejection and loss seem to emerge in this cluster.

Furthermore, in order to reach the desired child, the patients are willing to accept any cost, unconcerned about their health and the balance of their romantic relationship (Cluster 4). Their desire seems to be strongly “feminine,” extremely focused on the mother-child relationship, leaving in the background the male desire and the couple’s goal. Starting an ART procedure means that the physician, with his/her reproductive techniques, is symbolically placed in the middle of the couple’s relationship. This could create a triangular relationship where the husband may acquire a marginal role, excluded from the strong bond that can arise between the professional and the woman, who is the main subject of infertility treatments (Mann, 2014). Paradoxically, this could enhance women’s sense of loneliness. This study, indeed, shed light on feelings of loneliness and guilt experienced by these women, which totally devote themselves to the use of impersonal reproductive techniques and leave their couple relations in the background in order to reach the purpose of getting pregnant. These results suggest the usefulness of deepening the controversies concerning the marital relationship. In some cases, the cohesion of the couple favors the bond with the future child since the prenatal period (Busonera, Cataudella, Lampis, Tommasi, & Zavattini, 2017; Cataudella, Lampis, Busonera, & Marino, 2016); in other cases, childbirth is reported as a stressful event, producing a significant decline in marital quality (Castellano, Velotti, Crowell, & Zavattini, 2014).

The three identified factors seem to give a synthetic idea of the emotional functioning of the women involved in this study. ART treatment represents an emotional climax where passion and desire, on the one hand, and fear and uncertainty, on the other hand, are confronted (Factor 1). Women emotionally point to the relational context as their main resource to find support, both affective and technical/professional (Factor 3). ART treatment will decide their destiny: to belong to the world of the mothers, or to be unfortunate outcasts (Factor 2).

Overall, some limitations need to be accounted. The generalizability of the study is limited because these findings are related to a small sample (*n* = 17), comparable to a case study (Langher et al., 2017) and all subjects came from the same ART clinic. Another limitation of this study is that it is focused on the women’s perspective of the problem, and did not consider the men’s point of view, nor the couples’ perspective. Further studies should take into consideration men’s perspective in order to better understand the marital relationship dynamics as well as the influence of men’s desires and emotions about the choice of undergoing an ART procedure. Despite these limitations, this research study allows us to deeply investigate the emotions of the patients interviewed, namely the feelings they associated with their condition of infertility, the therapeutic procedures, and the awaited maternity. Our study may have an added value for its in-depth view of the emotional dynamics that characterize patients with infertility problems. In addition to the sense of sacrifice, the pursuit for social inclusion and the feeling of uncertainty about the future (Benyamini et al., 2005; Castellano et al., 2011; Lavender, Logan, Cooke, Lavender, & Mills, 2015; Verhaak et al., 2005), a lesser-known side of the desire for motherhood emerges in our research. Patients with infertility problems do not just seem to suffer from the great wound of infertility, but they are also strongly determined and resolute to reach their personal goal (the child), for which every relationship, including that with one's own body, is endangered. Therefore, this research study also contributes to highlighting the strong-willed and assertive features of these infertile women, which are not considered in many studies that describe them predominantly as suffering and distressed victims of infertility.

### Conclusion

The present research study shows the strong emotional investment on motherhood by women undergoing ART treatment. This makes them particularly demanding towards the fertility care staff, given their very high expectations. Analyzing their emotions could help better understanding the therapeutic relationship between infertile patients and reproductive healthcare professionals. Consequently, this could help to prevent burnout in staff members constantly confronted with highly emotionally demanding patients. The burden of witnessing the patients’ suffering due to their condition of infertility, their strong desire for parenting and determination can represent a source of high emotional pressure for the staff members, deemed as the only ones responsible for the fulfillment of the great dream of biological parenthood. For these reasons, a multidisciplinary approach, involving a psychological/medical staff to treating infertility, could benefit both patients and reproductive healthcare professionals. The participation of a psychologist in the clinical team would allow patients to be globally accompanied in their therapeutic experience and reproductive healthcare professionals to be relieved of the high emotional overload in their therapeutic relationship with patients with infertility problems, thus facilitating their clinical practice. Therefore, a multidisciplinary team approach that includes gynecologists, nurses, midwives, embryologists, psychologists as well as other professionals could improve the quality of the reproductive healthcare services offered to the patients suffering from infertility.

This study also have revealed a desire for parenthood strongly “feminine,” extremely focused on the mother-child relationship, leaving in the background the male desire and the couple’s goal. Some pieces of research in Taiwan examined the relationships of mothers who had naturally conceived and mothers who had artificially conceived with their newborn infants (Chen, Chen, Sung, Kuo, & Wang, 2011). This study found that mothers who had received infertility treatments in order to get pregnant had stronger bonds with their infants than mothers who had naturally conceived. Thus, it is possible that the partner feels emotionally excluded from the mother-child relationship, with consequent negative impact on the marital satisfaction of the couple. Traditionally, we know that the birth of the child (especially the first one) may represent a particularly critical moment for the couple: the sudden transition from a “dyad” to a “triad,” the so-called “birth of a family,” could indeed endanger the romantic balance between the partners (Cowan et al., 1985). Particularly, women’s high emotional investment on the pregnancy and on the newborn could represent a risk factor for the emotional stability of the couple. Some studies furthermore suggest that there is a significant association between parents’ dyadic relationship quality and the level of children’s behavioural problems. A more supportive relationship between the partners seems to be associated with fewer potential behavioural problems in children (Goldberg & Carlson, 2014; Harold & Lieve, 2012). Consequently, a low quality of romantic dyadic relationship may negatively impact on the children’s well-being and development. Moreover, the literature already affirms that fathers play a particularly important role in the child’s development, especially in terms of children’s openness to the world (Cabrera, Volling, & Barr, 2018; Paquette, 2004). A “closed” mother-child relationship can therefore circumscribe the role of the father in the development of the child, making it as secondary and marginal. Considering the strong emotional investment on motherhood, women who conceive with ART might also feel more constrained about actually expressing ambivalence or regrets than those conceiving naturally. They might also not feel entitled to complain or seek additional affective and social support.

In conclusion, we claim the importance of introducing early individual and couple psychological intervention into the centers of Assisted Reproductive Technology. We believe, in fact, that analyzing and addressing these complex emotional dynamics may have positive short-term/long-term effects on women, on the couple and on the relationship with the future newborn.
